# A feasibility study of activity tracking devices in pregnancy

**DOI:** 10.1186/s12884-019-2557-3

**Published:** 2019-11-04

**Authors:** Michelle A. Kominiarek, Lauren C. Balmert, Hallie Tolo, William Grobman, Melissa Simon

**Affiliations:** 1 Department of Obstetrics and Gynecology, Division of Maternal-Fetal Medicine, Northwestern University Feinberg School of Medicine, 250 East Superior Street, Suite 05-2175, Chicago, IL 60611 USA; 2 Department of Preventive Medicine, Division of Biostatistics, Northwestern University Feinberg School of Medicine, Chicago, IL USA; 3 Department of Obstetrics and Gynecology, Northwestern University Feinberg School of Medicine, Chicago, IL USA

**Keywords:** Pregnancy, Physical activity, Prenatal care, Feasibility study

## Abstract

**Background:**

We aimed to evaluate the feasibility of using an activity-tracking device (ATD) during pregnancy and compare self-reported to ATD-calculated energy expenditure in a 2-phase study.

**Methods:**

(Phase 1) Twenty-five pregnant women were asked about exercise, computer use, smartphone ownership, and ATD attitudes. Descriptive statistics were reported. (Phase 2) Women ≥18 years, smartphone owners, < 16-weeks gestation, and without exercise restrictions were approached to participate in 2016–2017. Women received instructions to wear and sync the ATD daily. We assessed protocol adherence and satisfaction via surveys at 36-weeks and used mixed models to assess the relationship between gestational age and ATD data. Energy expenditure from the Pregnancy Physical Activity Questionnaire (PPAQ) was compared to ATD-calculated energy expenditure.

**Results:**

(Phase 1) Walking was the most common exercise; 8% did not perform any activity during pregnancy. All women had internet access and owned a smartphone. Women stated they would wear the ATD all the time during a pregnancy (88%), with the intent to improve their health (80%). (Phase 2) The characteristics of the 48 women were: pre-pregnancy BMI 28, 62% non-Hispanic black, 62% multiparas. Of the 18 women who completed the 36-week survey, only 56% wore the ATD daily, 33% had a lost or broken ATD, and 17% had technical problems; however, 94% enjoyed wearing it, 94% would recommend it to a pregnant friend, and 78% thought it helped them reach activity goals. According to ATD data, the median number of active days was 41 (IQR 20–73) and the median proportion of active days out of potential days was 22% (IQR 11–40). As gestational age increased, mean log steps decreased, active minutes decreased, and sedentary hours increased in unadjusted and adjusted models (*P* < 0.05 all comparisons). There were no differences in mean energy expenditure (MET-h/week) estimated by PPAQ or ATD data at 28 weeks gestation [212 (22–992 range) vs. 234 (200–281 range), *P* = 0.66] and at 36 weeks [233 (86–907 range) vs. 218 (151–273 range), *P* = 0.68]).

**Conclusions:**

Women reported high motivation to wear an ATD and high satisfaction with actually using an ATD during pregnancy; however adherence to the study protocol was lower than expected and ATD technical problems were frequent.

## Background

Pregnancy is a time when women may be motivated to improve their health behaviors. As such, it is often considered the optimal time to intervene for issues related to eating habits and physical activity so as to prevent excessive gestational weight gain (GWG). In fact, the American College of Obstetricians and Gynecologists recommends that women engage in 30 min of moderate intensity exercise daily during pregnancy to maintain physical fitness, manage weight, and reduce the risk for gestational diabetes [1]. A meta-analysis of 49 randomized controlled trials with 11,444 women reported that diet or exercise interventions during pregnancy reduced the frequency of excessive GWG by 20% (relative risk [RR] 0.8, 95% CI 0.73–0.87) [2]. The exercise interventions reported in these studies were typically of moderate intensity and involved regular walking, dance or aerobic classes. Even though health behavior interventions during pregnancy show promising results with respect to GWG, many women continue to experience excessive GWG [3]. As such, further study is still required regarding health behavior interventions during pregnancy.

For an intervention for exercise or physical activity to be effective during pregnancy, it needs to consider the barriers that accompany this complex life stage such as physical and physiological adaptations (e.g., increased weight and fatigue), women receiving conflicting advice about the safety of exercise during pregnancy (e.g., from family, friends, and social media), and women prioritizing other family members’ health over their own. Examples of flexible and dynamic components include text messaging and m-Health apps (apps), which can potentially supplement an intervention that focuses on moderate intensity activity such as walking and facilitate engagement and retention. Self-monitoring, which is frequently cited as an important construct for long-term behavior change, can be built into app technology [4]. Reproductive age women are known to be frequent users of the internet, social media, and smartphone apps [5].

Activity tracking devices (ATD) can assess physical activity by providing data about steps taken, distance traveled, and energy expenditure or calories burned. We previously reported on the feasibility of ATD use during pregnancy and found high satisfaction with ATD use, but difficulties with protocol adherence [6]. Women in the prior study participated in a group prenatal care model where social support could have been a motivator to improve health behaviors including physical activity during pregnancy [6]. Another study found mixed results in terms of the association between ATD and health behaviors with stronger influences noted in the beginning of pregnancy, but less of an influence in the long-term [7]. Still, there are very few studies that have evaluated the feasibility of ATD use in pregnant women. Lastly, a woman’s perception of her activity during pregnancy may vary from objective measurements and we are not aware of studies that have compared self-reported to ATD calculated  activity. The purpose of this study was three-fold [1]: To evaluate the attitudes, beliefs, and opinions of pregnant women regarding the use of ATD for a future pregnancy (Phase 1) [2]; To evaluate the feasibility of using ATD in traditional prenatal care (Phase 2); and [3] To evaluate ATD data during pregnancy and compare self-reported to ATD calculated energy expenditure (Phase 2). We hypothesized that participants would express interest in using an ATD during a future pregnancy, wear the ATD for more than 80% of the time from enrollment until delivery, and that fewer than 10% of participants would report major issues or technical difficulties with the ATD. We also hypothesized that there would be differences in self-reported and ATD-calculated energy expenditure.

## Methods

Phase 1: To understand the acceptability of a study that involves ATD in pregnancy, we surveyed a convenience sample of 25 women over a two week period in October 2015 from a clinic that primarily serves low-income minorities in order to determine if women would participate in a study of actual ATD use during pregnancy. Inclusion criteria were pregnancy and English speaking. Participants completed a 5-page, 30-question face-to-face survey administered by one of the authors in a private room in the clinic while they were waiting for their prenatal care appointment after informed verbal consent was obtained. Survey questions included demographics and characteristics (age, self-reported race, gravidity, education, marital status, insurance status, gestational age, and current body mass index [BMI]). Other questions included types and frequency of exercise during pregnancy, prior activity monitoring methods, use of computers and internet, smartphone ownership, and attitudes towards ATD during pregnancy, but a self-assessment of daily activity with a validated survey was not performed. Participants were paid $10 in cash at the completion of the survey. Descriptive statistics were reported. The Northwestern University Institutional Review Board (IRB) considered this Phase 1 study exempt.

Phase 2: Subsequent to the completion of the survey assessment in Phase 1, a different group of pregnant women who were enrolled in prenatal care at the same clinic were approached at their prenatal visits and asked to participate in a study about “activity monitoring devices and pregnancy” from 2016 to 2017. Other inclusion criteria were English or Spanish speaking, ≥ 18 years old, personal ownership of a smartphone, and gestational age < 16 weeks. Exclusion criteria were pre-gestational diabetes or restrictions or inability to exercise, defined as at least 30 min of walking per day. These criteria were similar to the prior study of participants who participated in a feasibility study of ATD from a group prenatal care model during the same time period [6], but we opted to report the studies separately because the participants were recruited from separate sites, had differing demographics, and differing prenatal care models (traditional vs. group prenatal care), all of which could have been associated with ATD use and study protocol adherence.

Participants picked a wrist Fitbit Flex™ (i.e., the ATD) of their color preference after informed written consent was obtained. The set-up for the ATD has been previously described and is briefly summarized here [6]. Research team members registered the participants’ ATD online and created user accounts which were available in both English and Spanish. Participants also received a 10-min in-person counseling session on safe exercises during pregnancy [1, 8]. Individualized step count goals were encouraged, though 10,000 steps per day was presented as one goal as it was the default step count goal of the Fitbit device and considered a reasonable goal for healthy adults [9–11]. Steps, active minutes, and sedentary hours were wirelessly transmitted via cellular and Bluetooth technology and plotted on a graph in the ATD app. Fitbit technology uses metabolic equivalents (METs) to calculate active minutes, defined as any activity ≥1.5 METs. We did not replaced lost, stolen, or broken ATD. The research team was available for technical support on an as-needed basis.

Details of the study design and statistical analyses are also described in the prior study [6]. Briefly, participants completed several surveys including demographic characteristics and personal technology use (baseline), the Pregnancy Physical Activity Questionnaires (PPAQ) [12] (baseline, 28, and 36 weeks gestation), health behavior changes and satisfaction (36 weeks gestation) [13]. Each participant was counseled to wear the ATD continuously and only remove it when it was at risk for damage (e.g., swimming, bathing, etc.) or being charged. It was expected that the ATD would be synced every day and charged every 5 days. The participants kept the ATD at the end of the study (delivery date), at which time the research account was deactivated. Reasons for drop outs, technical problems, and adherence to the ATD were summarized. The proportion of active days, defined as a minimum count of 1000 steps per day, out of potential days and number of participants wearing the ATD for at least seven consecutive days were reported. ATD data (mean daily steps, active minutes, and sedentary hours) were summarized for the first full week of use.

Trends in steps, active minutes, and sedentary time by gestational age were visually assessed and mixed models assessed the trajectory of the three outcomes during pregnancy [14]. A fixed effect for gestational week and a random effect for participants to account for correlations of measurements within participants were included in the models. Adjusted models considered covariates such as age, ethnicity, pre-pregnancy BMI, and education. Paired t-tests or Wilcoxon signed rank tests assessed changes in PPAQ responses over time. Mean MET-h/week for the second and third trimesters also were calculated from ATD data. Wilcoxon signed rank tests were used to compare energy expenditures between the PPAQ and ATD data. Lastly, the equality of variance between the two measures was assessed with Pitman’s test and Spearman correlations.

A *p*-value < 0.05 was considered statistically significant. Analyses were performed with SAS, version 9.4. The Northwestern University IRB approved the Phase 2 portion of the study and informed written consent was obtained from all participants in Phase 2.

## Results

Phase 1: Of the 27 women approached to participate, only two declined, primarily due to time constraints. Of the 25 participants, all had a high school degree, most were minorities (84% either Hispanic or non-Hispanic black) with a mean (± standard deviation) age of 29 ± 6 years, and a median gravidity of 3 (IQR 2–4). The majority were unmarried (84%) and had Medicaid insurance (96%). The mean gestational age was 26 ± 5 weeks and the mean BMI at the time of the survey was 33 ± 8 kg/m^2^ with > 50% of participants having a BMI > 30 kg/m^2^.

The most common form of activity was walking (80%) followed by activities around their home and/or job (52%). Only 40% of women aimed to meet the daily exercise recommendations [1] and 12% had no intention to exercise during pregnancy. More than half of the participants were not tracking their activity during pregnancy prior to the survey, but of those who were, the most common format was a droid or iPhone app (32%). Only one participant had a Fitbit device and most had never used an ATD, though many were aware of the ones that were already on their smartphones. There was universal use of the internet and computer access and 100% of participants had a smartphone. Most participants stated they would not need any help (88%) with the initial start-up and follow-up required to use the ATD, and that they would wear the device all the time (88%). The preferred location to wear the device was on the wrist (96%) and the two favorite colors were purple and pink. Most women would chose to wear the device because they were interested in improving their health (80%). Participants were also interested in knowing their daily steps and how their activity varies day-by-day. Most women (68%) could not think of a reason not to wear the device. At completion of the survey, the majority of the women voluntarily stated that they were interested in obtaining such a device.

Phase 2: Given the positive findings from Phase 1, we proceeded with Phase 2. Of the 100 women approached to participate in the study, 50 declined. The most common reasons for declining were lack of interest or the request for additional time to consider the study requirements without any subsequent contact during the recruitment period. Of the 50 women enrolled in the study, two were lost to follow-up (e.g., did not complete initial surveys or never used the ATD), two requested to drop out of the study and returned the ATD, and 4 had miscarriages < 20 weeks. Analyses included any non-missing data for the initial 48 women.

Most participants identified as non-Hispanic black (61.9%) and were multiparas (62.5%).(Table 1) Based on the pre-pregnancy BMI, 43.8% were either overweight or obese. More than 85% reported using the internet daily and a high level of comfort using computers. Only 9.5% exercised daily prior to pregnancy. The mean number of prenatal care visits was 10.7 ± 3 (*n* = 42).

**Table 1:** Maternal Demographics and Characteristics for 48 participants

Variable	Response
Age, years (mean ± SD)	28.0 ± 5.4
Race-Ethnicity, *n*(%)^a^	
Asian American	0 (0.0)
Black/African American Hispanic/Latino	26 (61.9) 12 (28.6)
White	4 (9.5)
Education, *n*(%)^a^	
Grades 9–11	1 (2.4)
High school graduate/GED	10 (23.8)
Some college/technical school	19 (45.2)
Four year college degree or more	9 (21.4)
Missing	3 (7.1)
Health insurance, *n*(%)^a^	
Medicaid or Medicare	35 (83.3)
Private Insurance	3 (7.1)
Other	1 (2.4)
Missing	3 (7.1)
Employed outside of the home for a salary, *n* (%)^a^	
Yes	20 (47.6)
No	19 (45.2)
Missing	3 (7.1)
Marital status, *n* (%)^a^	
Married	11 (26.2)
Single	19 (45.2)
Living with partner, but not married	9 (21.4)
Missing	3 (7.1)
Multipara, *n* (%)^a^	30 (62.5)
Gestational age at enrollment, weeks (mean ± SD)	13.1 ± 2.2
Trimester at enrollment, *n* (%)	
First	20 (41.7)
Second	28 (58.3)
Pre-pregnancy BMI (mean ± SD) (*n* = 38 women)	28.4 ± 9.1
Pre-pregnancy BMI, *n* (%)(*n* = 38 women)	
Underweight	10 (20.8)
Normal	17 (35.4)
Overweight	9 (18.8)
Obese	12 (25.0)
History of regular cigarette use, *n* (%)^a^	
Yes	7 (16.7)
No	35 (83.3)
Self-reported daily internet use, *n* (%)^a^	36 (85.7)
Self-reported “very comfortable” using a computer and/or the internet, *n* (%)^a^	39 (92.9)
Type of smartphone owned, *n* (%)^a^	
iPhone	24 (57.1)
Droid	16 (38.1)
Other	1 (2.4)
Missing	1 (2.4)
“Before pregnancy, how much did you exercise?”, *n* (%)^a^	
Not at all	9 (21.4)
Occasionally	11 (26.2)
Once a month	2 (4.8)
Once a week	5 (11.9)
More than 1 time a week	11 (26.2)
Everyday	4 (9.5)

^a^ Subset of analytic cohort completing baseline survey *n* = 42

*GED* General Equivalency Development, *BMI* Body mass index

Regarding the 36-week adherence and satisfaction surveys, of the 18 participants who completed surveys, only 55.6% reported wearing the ATD all the time. (Table 2) The most common reasons for not wearing the ATD were a lost or damaged device, not being able to wear it while working, or packing it in a box when they moved residences. Difficulties that participants commonly reported were related to syncing the tracker with the app, yet the majority enjoyed wearing the ATD (94.5%), would recommend it to a pregnant friend (88.9%) and thought that it helped them reach their activity goals (77.8%). However, fewer thought that being in the study helped them eat more healthfully (44.4%) or reach their weight gain goals (33.3%).(Table 3).

**Table 2:** Self-reported adherence and changes in health behaviors based on surveys at 36 weeks (*n* = 18)

Variable	Response at 36 weeks *n*(%)
Adherence	
“How often are you wearing the ATD?”	
All the time	10 (55.6)
A few hours a day	1 (5.6)
Only when I’m awake	1 (5.6)
A few days a week	1 (5.6)
Other	4 (22.2)
Missing	1 (5.6)
“I have difficulties wearing the ATD because”: ^a^	
Lost or stolen device	6 (33.3)
Concern that it would get damaged if it got wet	1 (5.6)
Broken device	3 (16.7)
Other reasons (e.g., forget to charge or wear, moved residence, personal problems)	4 (22.2)
“I had the following problems with the ATD or app.”^a^	
Internet connection problems	1 (5.6)
Did not like the website	1 (5.6)
Did not like wearing Fitbit tracker	1 (5.6)
Difficulty getting Fitbit tracker to sync with website	2 (11.1)
Other technical problems with Fitbit tracker	3 (16.7)
Lost or broken Fitbit tracker or charger	6 (33.3)
Other	6 (33.3)
“What were the benefits of wearing an ATD for you?“^a^	
I knew the number of steps I took per day	12 (66.7)
I learned how my activity varies each day	10 (55.6)
I improved my health by tracking my activities and goals	5 (27.8)
Health Behavior Changes	
“How much are you exercising since before pregnancy?”	
More Often	1 (5.6)
About the Same	13 (16.7)
Less Often	14 (77.8)
“Physical activity that makes me breathe harder is ok at any time during pregnancy.”	
Strongly agree	5 (27.8)
Agree	7 (38.9)
Disagree	6 (33.3)

^a^Categories are not mutually exclusive, so percentages do not sum to 100

*ATD* Activity tracking device

**Table 3:** Self-reported satisfaction based on surveys at 36 weeks (*n* = 18)

Satisfaction Questions *n*(%)	Strongly Agree	Agree	Disagree	Strongly Disagree
I found the Fitbit website and dashboard easy to navigate.	8 (44.4)	10 (55.6)	1 (3.3)	1 (3.3)
I found the smartphone Fitbit app easy to use.	15 (83.3)	3 (16.7)	0	0
I enjoyed wearing the Fitbit.	10 (55.6)	7 (38.9)	1 (5.6)	0
I would recommend the Fitbit to pregnant friend.^a^	11 (61.1)	5 (27.8)	1 (5.6)	0
Being in this study helped me eat healthier.^a^	2 (11.1)	6 (33.3)	7 (38.9)	0
Being in this study helped me reach my activity goals.^a^	4 (22.2)	10 (55.6)	2 (11.1)	1 (5.6)
Being in this study helped me reach my weight gain goals.^a^	2 (11.1)	10 (55.6)	2 (11.1)	2 (11.1)
I am satisfied with my weight gain this pregnancy.^a^	7 (38.9)	7 (38.9)	2 (11.1)	1 (5.6)

^a^*n* = 17 due to missing data

According to the pairwise comparisons of PPAQ self-reported activity during the three time periods, there were significant differences between the 28 week and 36 week surveys for the 13 participants who had completed both surveys for total activity (28 week: 197.7 MET-h/week, interquartile range [IQR] 107.5, 439.5 vs. 36 week: 237.9 MET-h/week, IQR 148.5, 393.9; *P*-value = 0.02) and sedentary time (28 week: 12.6 MET-h/week, IQR 7.4,29.4 vs. 36 week: 24.2 MET-h/week, IQR 7.4, 44.8; P-value = 0.03.(Table 4).

**Table 4:** Comparison of self-reported activity from the Pregnancy Physical Activity Questionnaire at three time points during pregnancy

Median activity in MET-h/week (IQR)	Baseline (*n* = 43)	28 weeks (*n* = 24)	36 weeks (n = 18)	P-value
Total activity	269.2 (171.2, 385.4)	197.7 (107.5, 439.5)	237.9 (148.5, 393.9)	0.58^a^ 0.44^b^ 0.03^c^
Sedentary activity	17.9 (7.4, 44.8)	12.6 (7.4, 29.4)	24.2 (7.4, 44.8)	0.42^a^ 0.80^b^ 0.03^c^

^a^Comparisons for 24 participants who completed baseline and 28 weeks survey

^b^Comparisons for 18 participants who completed baseline and 36 weeks survey

^c^Comparisons for 13 participants who completed 28 weeks and 36 weeks survey

MET metabolic equivalents

IQR interquartile range

**Fig. 1:**
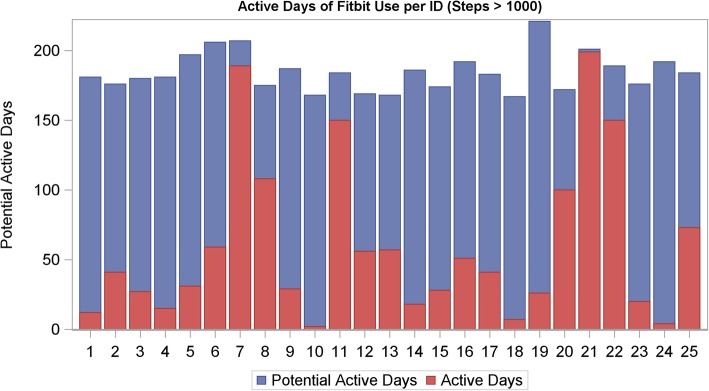
Active days of activity tracking device use, defined as at least 1000 steps/day (red bars) as a proportion of total potential active days (blue bars) from date of enrollment to date of delivery for 25 participants who did not report permanent activity tracking device or cellphone problems (e.g., lost or broken device or charger, loss of cellphone access). The median proportion of active days was 22% (IQR 11.0,40.0)

**Fig. 2:**
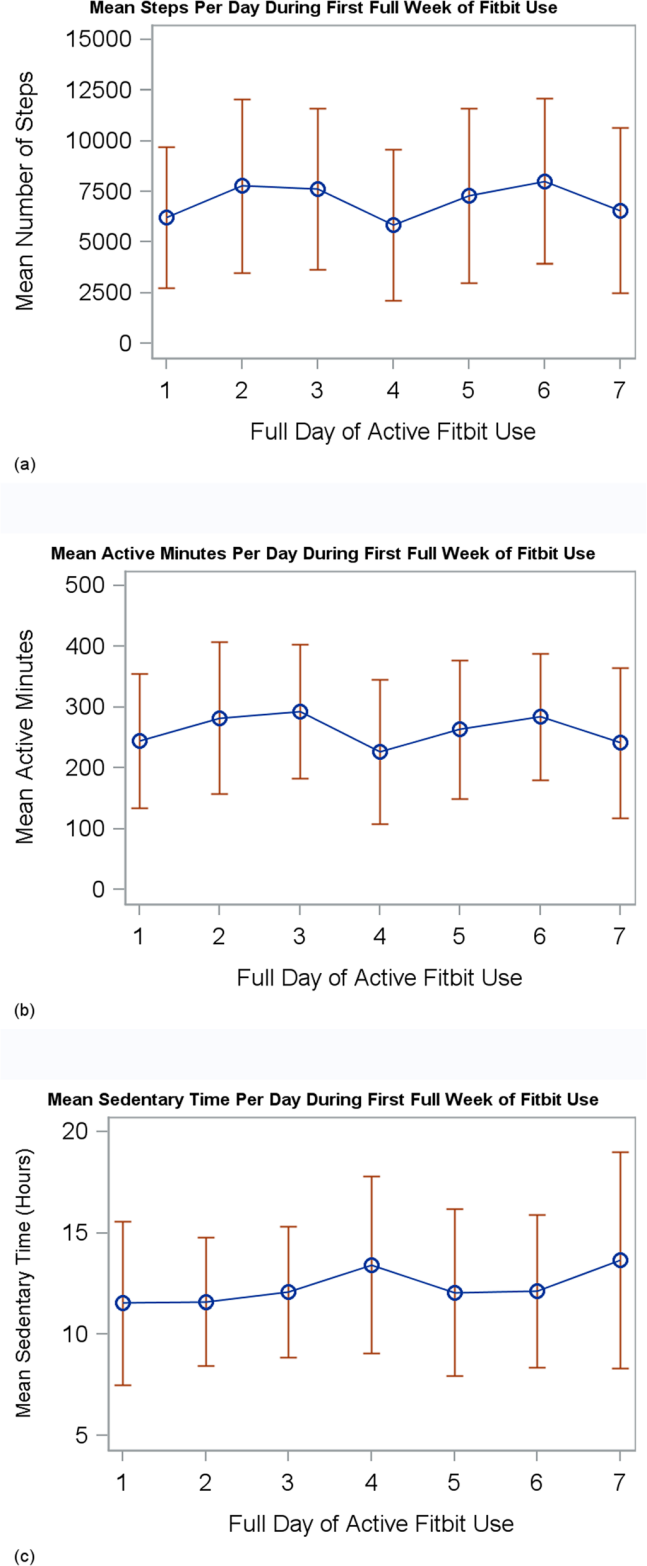
Mean (blue bars) and range (red bars) of (**a**) steps, (**b**) active minutes, and (**c**) sedentary hours for 29 participants who wore the activity tracking device consecutively for the first seven days. The mean steps per day were 7050 (range 2286–15,133), active minutes per day were 262 (range 100–395), and sedentary hours per day were 12.4 (range 8.5–19.3)

In the analysis of the ATD data, the median number of active days was 41.0 (IQR 20.0, 73.0) and the median proportion of active days was 22% (IQR 11.0, 40.0) (Fig. 1) for a sample of 25 participants (i.e. excluded those who lost the ATD or charger and/or encountered unresolved syncing or cellphone difficulties). The median number of days women had the potential to wear the ATD was 183 (IQR 175,192). For the 29 participants who wore the ATD consecutively for the first full seven days of enrollment, the mean steps per day during the first week were 7050 (range 2286–15,133), active minutes per day were 262 (range 100–395), and sedentary hours per day were 12.4 (range 8.5–19.3).(Fig. 2).

**Fig. 3:**
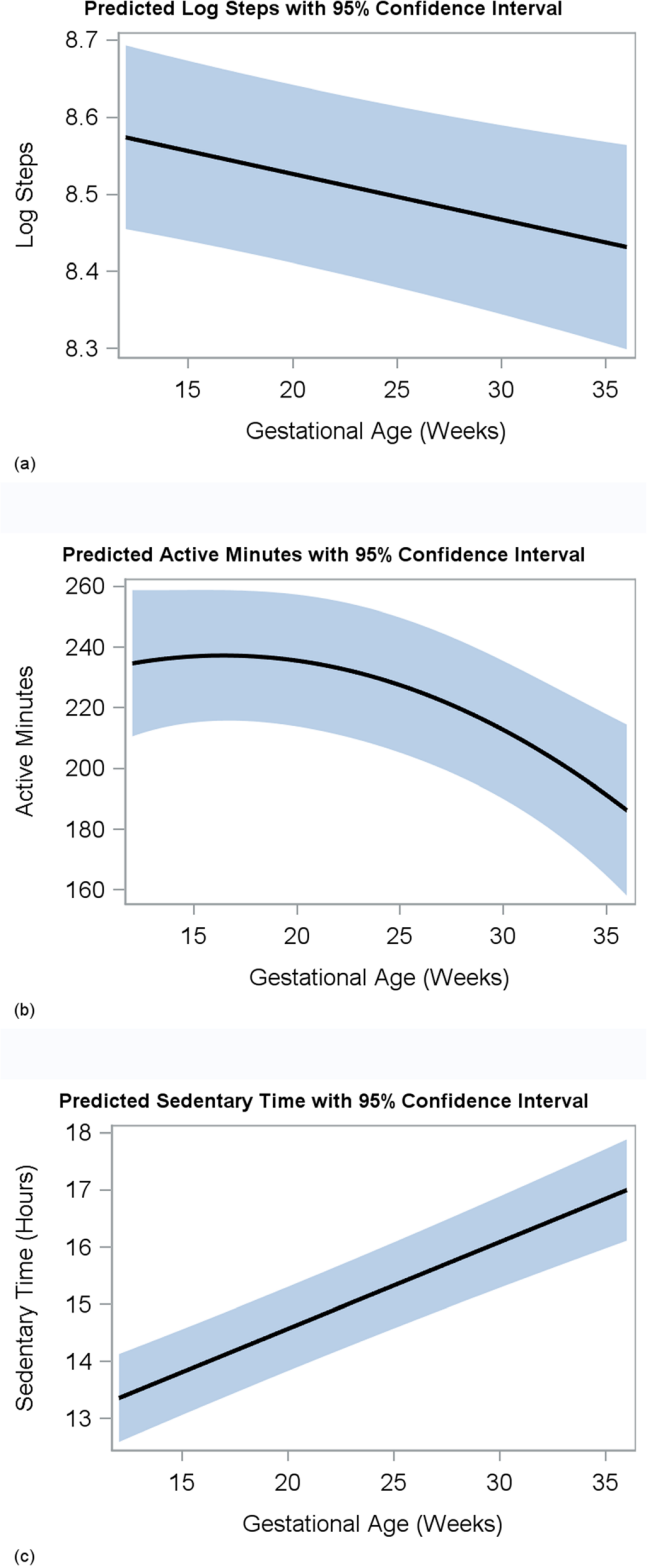
Longitudinal modeling for activity tracking device data with predicted (**a**) logarithmic steps (β Gestational week = − 0.006, *P*-value = 0.004), (**b**) active minutes (β Gestational week^2^ = − 0.133, P-value = 0.03), and (**c**) sedentary hours (β Gestational week = 0.15, *P*-value < 0.001) as denoted by black lines with 95% CI (shaded area) plotted against gestational age with *p* < 0.05 for change over time for all comparisons

As gestational age increased, mean log steps decreased (β Gestational week = − 0.006, *P*-value = 0.004, Fig. 3a) and sedentary hours increased (β Gestational week = 0.15, P-value < 0.001, Fig. 3c) according to the longitudinal models. A significant quadratic relationship was found for mean active minutes indicating a steeper decline as gestational age increased (β Gestational week^2^ = − 0.133, *P*-value = 0.03, Fig. 3b). The findings were still statistically significant after adjusting for age, ethnicity, pre-pregnancy BMI category, and education. There were no significant differences in median energy expenditure (MET-h/week) recorded by PPAQ or ATD data at 28 weeks [*n* = 23, 212 (22–992 range) vs. 234 (200–281 range), *P*-value = 0.66] and at 36 weeks [*n* = 14, 233 (86–907 range) vs. 218 (151–273 range), P-value = 0.68], but variances of these measures differed at both 28 weeks and 36 weeks (P-value < 0.001), with variances of self-reported PPAQ activity being much larger. (Table 5).

**Table 5:** Comparison of median energy expenditure between the Pregnancy Physical Activity Questionnaire and activity tracking device

Median energy expenditure (MET-h/week)	Self-report^a^ Median (range)	Activity Tracking Device Median (range)	*P*-value ^b^	*P*-value ^c^
28 weeks (n = 23)	212 (22–992)	234 (200–281)	0.66	< 0.001
36 weeks (*n* = 14)	233 (86–907)	218 (151–273)	0.38	< 0.001

^a^MET-h/week derived from self-reported activity from Pregnancy Physical Activity Questionnaire

^b^*P*-value from Wilcoxon signed rank test

^c^*P*-value from Pitman’s test for equality of variance with Spearman correlation

MET metabolic equivalents

## Discussion

In this two phase study, we aimed to first determine the attitudes, beliefs and opinions of pregnant women regarding the use of ATD in a future pregnancy and then determine the feasibility of actually using ATD during pregnancy in a separate cohort of women. When asked about ATD use during a future pregnancy, women reported that an ATD was acceptable, that they believed they would not have issues with understanding the technology to set-up the ATD, that they had nearly universal access to internet and smartphones, and that that they would use an ATD daily (Phase 1). When given an ATD, most participants reported satisfaction with the product and the information it provided, and noted that they would recommend the ATD to others (Phase 2). Adherence to the Phase 2 study protocol was lower than expected based on the Phase 1 findings of 88% of women reporting that they would wear an ATD daily during pregnancy. Only half of the participants who completed the 36-week survey self-reported daily ATD wear, consistent with our prior study [6]. This finding was also substantiated in our ATD data of 25 participants (50% of total sample) who wore the ATD only 22% of possible days, on average. As in our previous study, we also found high reports of major issues or technical difficulties with the ATD. Some of the commonly reported adherence issues were related to a lost or broken ATD or charger or technical problems such as syncing issues. In longitudinal modeling, we found a statistically significant decrease in total activity and increase in sedentary time as pregnancy progressed.

Our findings have similarities and differences compared to prior reports. A review of 71 articles regarding electronic health developments in pregnancy also found that the majority (88%) of women owned a smartphone, and that as many as 98% used websites and pregnancy apps [15]. When using those sites and apps, “healthy lifestyle during pregnancy” was among the most frequently searched topics [15]. However, only a few studies have reported on actual ATD use in pregnancy. Huberty et al. also described similar trends in activity with a decline in activity and increase in sedentary time during pregnancy in 80 inactive pregnant women [14]. Grym et al. had greater compliance in their 20 nulliparas who wore an ATD 76% of potential days over a 7 month period, starting in the second trimester until the first postpartum month [7]. Similar to our studies, technical problems were common as 55% had issues related to device charging and syncing and 40% had issues with the wristband.

Of significant interest, we noted that self-reported total activity as assessed via the PPAQ increased from 28 weeks to 36 weeks whereas ATD step counts declined with gestational age. These findings also need to be interpreted in the context that 77.8% of women reported that they were exercising less-often at the final survey compared to before pregnancy. Similar to our prior study, there was not a statistically significant difference in mean MET-h/week by self-report via PPAQ vs. ATD data and we did observe greater variation in PPAQ vs. ATD METs [6]. It is possible that the greater variation in the PPAQ values explains the lack of difference in comparisons between METs with the PPAQ and ATD. Since there is no recall bias with ATD measurements, it is also possible that the ATD has greater accuracy, but further studies are needed to study this hypothesis.

Our ATD data support findings from other studies of physical activity in pregnancy including a decrease in activity and increase in sedentary time as pregnancy progresses [16]; however the ATD offers a more contemporary approach to activity monitoring. Nevertheless, given that a high proportion of participants did not use the ATD according to the protocol, future trials that use an ATD will need to consider intermittent ATD use (e.g., 1 week periods) or monitor activity with apps that are already part of their smartphone to enhance compliance.

Participants who opted out of the study after enrollment and returned the ATD did so for personal, not technical reasons. Women encounter many challenges when they attempt to make lifestyle changes, especially during pregnancy. For example, participants may have personal beliefs or receive conflicting messages from other providers or support systems about the potential risks of exercise in pregnancy. Given that a third of participants at 36 weeks of gestation disagreed with the statement, “Physical activity that makes me breathe harder is ok at any time during pregnancy”, we suspect these conflicts with professional advice play an important role in a woman’s choice to engage in physical activity during pregnancy. We also noted that women reported that changes in routines (e.g., holidays, moving residences) made it difficult to remember to wear the ATD. Focus group interviews with women after use of the device may have elucidated the specific barriers that women encountered when using the ATD.

We acknowledge limitations to this study. Generalizability cannot be assured, as these findings were performed at a single location with a small sample size. Women were recruited for this study during the same time period as our prior publication, but we a-priori chose to present the findings from this study in a separate report because of the differences in demographic characteristics (e.g., 62% non-Hispanic black in current study and 80% Hispanic in prior study) and differences in prenatal care model (traditional vs. group), which could have influenced the overall study findings [6]. Similar to the prior study, many women did not complete all the study procedures and there could be selection bias in the reporting of the 36-week survey findings from only 18 women. Whether our findings would be similar for devices other than the Fitbit is unknown. We opted to use the Fitbit model for our ATD because it had the highest rating for validity in other studies, but to our knowledge, there had been no formal validity testing of Fitbits in pregnant women prior to the initiation of our study [17–19].

## Conclusion

Women reported high motivation to wear an ATD and high satisfaction with actually using an ATD during pregnancy; however adherence to the study protocol was lower than expected and ATD technical problems were frequent.

Further study is required to determine best practices to engage women in physical activity and reduce sedentary behaviors in pregnancy, especially in the third trimester. Future research should also investigate barriers and facilitators to ATD use in pregnancy and determine whether ATD use or other types of activity tracking in physical activity interventions is associated with improved obstetric outcomes besides GWG, such as gestational diabetes, pregnancy related hypertension, and Cesarean delivery. ATD have other features such as sleep quality and duration and heart rate monitoring that can also be evaluated with respect to physical activity and obstetric outcomes in future studies.

## Data Availability

The datasets used and/or analysed during the current study are available from the corresponding author on reasonable request.

## Abbrviations

RR: Relative risk
IRB: Institutional Review Board
IQR: Interquartile range
ATD: Activity tracking device
PPAQ: Pregnancy Physical Activity Questionnaire
METs: Metabolic equivalents
BMI: Body mass index
GWG: Gestational weight gain
REDCap: Research Electronic Data Capture
